# Effects of bio-nano-selenium on wheat grain morphology, selenium transport enrichment and antioxidant enzyme activities

**DOI:** 10.3389/fpls.2025.1516005

**Published:** 2025-03-05

**Authors:** Sisi Huang, Yali Han, Ruilian Song, Xiaofang Wang, Yu Zhou, Hongmei Luo, Xifeng Ren, Kan Yu

**Affiliations:** ^1^ Institute of Agricultural Economy and Technology, Hubei Academy of Agricultural Science, Wuhan, China; ^2^ Institute of Agricultural Grain and Oil, Handan Academy of Agricultural Science, Handan, China; ^3^ College of Plant Science and Technology, Huazhong Agricultural University, Wuhan, China

**Keywords:** bio-nano-selenium, wheat, physiological indicators, grain morphology, agronomic traits

## Abstract

Selenium (Se) is an essential trace element for human health, but selenium deficiency is widespread worldwide. In this study, we investigated the effects of selenium uptake, grain morphology, and antioxidant enzyme activities in three wheat varieties, including Huamai 1168 (high gluten), Huamai 2152 (medium gluten), and Wanximai 0638 (low gluten), by foliar spraying of bio-nano-selenium at the early flowering stage of wheat. The bio-nano-selenium nutrient solution was a patented product of microbial fermentation (Patent No. 201610338121.6) independently developed by our team, with a pure selenium concentration of 5000 mg/kg. The results showed that the total selenium content in all the varieties increased by 1843.52%, and the organic selenium content increased by 2009.87%, with Huamai 1168 showing the highest total selenium and organic selenium content. After selenium treatment, CAT activity decreased in all varieties; POD and SOD activities showed a tendency to increase and then decrease; MDA and proline content increased; and GSH content fluctuated during the filling period. Overall, foliar spraying of selenium enhanced antioxidant enzyme activities and improved the plants’ ability to cope with environmental stresses. In terms of agronomic traits, bio-nano-selenium positively affected plant height (12.63% increase on average), effective spike number (17.24% increase on average), and spikelet number (17.81% increase on average), but had a limited effect on grain morphology. In addition, bio-nano-selenium not only increased soil nutrient content but also promoted the uptake of hydrolyzed nitrogen, effective phosphorus, fast-acting potassium, and sulfate in wheat. In summary, bio-nano-selenium is expected to be an effective tool for selenium biofortification of wheat, which not only significantly increases the selenium content of grains but also improves yields, stress tolerance, and fertilizer utilization, providing a potential solution to selenium deficiency through dietary solutions, while contributing to the sustainable development of agriculture.

## 1 Introduction

Selenium (Se) is one of the essential trace elements for humans and animals (Jones et al., 2017) and has pleiotropic effects, such as antioxidant and anti-inflammatory properties, production of active thyroid hormones, and regulation of sperm motility (Rayman, 2012). Studies have reported that Chinese residents are generally selenium-deficient, with an average selenium intake of only 43.3 μg d^-1^, significantly lower than the WHO-recommended 60 μg d^-1^ (Duan, 2018). Approximately 1 billion people worldwide have insufficient daily selenium intake (Zhou et al., 2019). When people’s dietary selenium intake falls below 40 μg d^-1^, symptoms of selenium deficiency can occur (Gupta and Gupta, 2017). Although selenium is crucial for human health, the body cannot synthesize selenium on its own and can only obtain it through the diet (Park et al., 2023). Organic selenium in plants mainly includes selenocysteine, selenomethionine, and others. Organic selenium is safer and more effective than inorganic selenium, being easily absorbed and utilized by the human body, with an absorption rate exceeding 81% (Rayman, 2008). The biological effectiveness of selenium in plants tends to be higher than that in animals, and plant-derived selenium often determines the selenium level in the food chain (Yang et al., 2021).

Selenium is a beneficial micronutrient for plant growth, with multiple physiological roles and antioxidant properties that play an important role in plant response to abiotic stress (Huang G. et al., 2020). Selenium has been shown to affect a wide range of plants, including tea (Liu K. et al., 2021), halibut (Nithyananthan et al., 2023), courgette (Alsamadany et al., 2023), rice (Ramos et al., 2023), maize (Guo et al., 2019), mung bean (Kamali-Andani et al., 2022), and wheat (Elkelish et al., 2019). These studies have shown that selenium plays a physiologically important role in plants, but the mechanisms of selenium uptake and response vary from plant to plant, as evidenced by variations in selenium accumulation patterns and antioxidant enzyme activities, among other factors. The cycling process of selenium in nature is relatively complex, and selenium in the soil is gradually converted from inorganic selenium to organic selenium after being absorbed by plants, and is eventually absorbed and utilized by the human body through the food chain (Gui et al., 2022). However, there are limitations in the utilization of natural selenium-enriched soils, such as uneven distribution or insufficient concentration of selenium, which limit the yield and quality of selenium-enriched agricultural products. Therefore, an increasing number of studies have focused on enhancing the selenium concentration in agricultural products through exogenous selenium supplementation, which is not only easy to control but also cost-effective and more efficient in production. Increasing crop selenium concentration through exogenous selenium supplementation to meet people’s daily selenium demand has become an effective way to solve the selenium deficiency problem (Xiao et al., 2023; Cai et al., 2023; Guo et al., 2019).

Cereal crops are particularly suitable for exogenous selenium fortification, as they are the main source of selenium supplementation for humans in most countries worldwide (Haug et al., 2009). Wheat grains provide a large amount of important nutrients to people; not only does the protein content far exceed that of other cereal crops, but it is also the cereal crop with the highest selenium enrichment capacity. Exogenous selenium fortification of wheat has been successful (Wang et al., 2020; Boldrin et al., 2016, 2018). About 63% of wheat in China is selenium-deficient, with the average selenium concentration in wheat grains being 64.6 μg kg^-1^, which is far from meeting the basic selenium requirements of the human body (Li et al., 2020). Residents who rely on wheat as a staple food are recommended to have a selenium concentration of 150-300 μg kg^-1^ in wheat grains (Lyons, 2010). Previous studies have demonstrated that exogenous fortification can significantly increase the selenium content in wheat grains, with the selenium-enrichment capacity varying across different wheat varieties (Wang et al., 2021; Lyons et al., 2005; Al-Ghumaiz et al., 2020).

However, most of these studies have used inorganic selenium sources, and there are fewer reports on the application of bio-nano-selenium in crops (Huang et al., 2023a, b). Compared with inorganic and organic selenium, nano-selenium, with its high particle dispersion, bioactivity, low acute toxicity, and high human tolerance, is a promising and emerging field in nanotechnology with broad application prospects (Bhattacharjee et al., 2019; Nikam et al., 2022). Currently, the application of nano-selenium in agriculture mainly focuses on improving plant productivity (Huang et al., 2023b, 2023; Liu et al., 2022; Zahedi et al., 2019) and biological control (Shi et al., 2023; Zhang et al., 2023). Based on the unique advantages of bio-nano-selenium sources, the differences in selenium uptake across wheat varieties, and the current research gaps regarding bio-nano-selenium in wheat, this study utilized a bio-nano-selenium nutrient solution (Patent No. ZL201610338121.6) (Liu et al., 2016; Huang et al., 2023a), which was independently developed by our team, for foliar spraying at the early blooming stage of wheat. The study aimed to investigate its effects on leaf antioxidant, grain morphology, selenium accumulation, yield, and soil in different wheat varieties, and to provid valuable references for the research on the production technology of high-yield, high-quality, organic selenium-rich wheat.

## 2 Materials and methods

### 2.1 Experimental materials and design

The three wheat varieties selected in this study were the high-gluten wheat variety Huamai1168, the medium-gluten wheat variety Huamai2152, and the low-gluten wheat variety WanXimai0638. These varieties are representative selenium-enriched wheat cultivars, screened through the application of a bio-nano-selenium nutrient solution in the wheat-growing areas of the middle and lower reaches of the Yangtze River in China (Huang S. et al., 2020). The bio-nano-selenium nutrient solution we used in the experiment was a patented product developed by our research team (Patent No. ZL201610338121.6), and the pure selenium content of this solution was ≥5000 mg L^-1^, and the average particle size of nano-selenium was 126 ± 0.5 nm. The experimental site was located at the Nanhu Experimental Farm of Hubei Academy of Agricultural Sciences in Wuhan City, Hubei Province (114°18’ E, 30°29’ N, elevation 30 m), with a soil pH of 6.29.The average rainfall in 2022 was 1,042.3 mm, which belongs to the year of partial dryness, and the growing season of wheat is from November to May of the following year.

The experiment was conducted using a completely randomised block design, with selenium treatment and control groups for each variety, each replicated three times, for a total of 18 plots. The flowering period of each plot was determined when more than 50% of the wheat plants were in flower, and wheat spikes within the plots were tagged on the day of flowering, with 120 spikes tagged per plot. The selenium treatment group was foliar sprayed on the 4th day of wheat flowering, with 29 mL of bio-nano-selenium nutrient solution applied per plot, equivalent to 145 mg of pure selenium. This application was converted to 4,500 mL of bio-nano-selenium nutrient solution per hectare, providing 23,000 mg of pure selenium, at a concentration of 30 mg L^-1^. The control group was sprayed with an equal amount of aqueous solution at the same time. Each plot had an area of 63 m² (25 m × 2.5 m), with a planting row spacing of 0.25 m. Other field management practices were consistent with the local wheat field management protocol.

### 2.2 Examination items and methods

On the 10th, 15th, 20th, 25th and 30th days after foliar spraying of the bio-nano-selenium nutrient solution, the saber leaves from five hanging spikes in each plot were collected. The samples were placed in ice packs immediately after sampling and stored at 4°C. The following parameters were measured: catalase (CAT) activity, peroxidase (POD) activity, superoxide dismutase (SOD) activity, malondialdehyde (MDA) content, reduced glutathione (GSH) content, and proline (Pro) content. The assay methods are described in **Supplementary Table S1**.

After full maturation of each material, 6 plants were randomly selected for harvesting, threshing and drying until the grain moisture content fell below 14%. Grain with pests and diseases were excluded. The morphological traits of the grain from each material were measured using the Wanshen SC-G Automatic Grain Analysis System. Measured traits included grain area (Ga, mm^2^), grain perimeter (Gp, mm), grain width (Gw, mm), grain length (Gl, mm), grain length-width ratio (Lwr), grain roundness (Gr) and grain diameter (Gd, mm), thousand grain weight (Tgw, g), and the factor from density (Ffd). The assay method is described in **Supplementary Table S1**.

After the test materials fully matured, 10 plants were randomly selected from each experimental plot to examine their agronomic traits. Eight trait parameters were measured for each plant: plant height (PH, cm), number of effective spike (ES), Length of main spike (LMS, cm), panicle neck length of main spike (LFPMS, cm), rachis internode length of main spike (RLMS, cm), spikelets on main spike (SMS), number of spikelets per plant (NSPP), and number of grains per plant (GNP). The average of the 10 measured values was taken as the phenotypic value of each trait in the plot. The assay methods are described in **Supplementary Table S1**.

Approximately 250 g of soil was collected at a depth of 20 cm below the surface in each plot planted with Huamai1168 using the 5-point sampling method. The samples were mixed well, dried, and then 500 g of soil was taken to examine the basic physical and chemical properties and total selenium content in the soil of both the selenium-treated and control groups. The basic physical and chemical properties of the soil were tested according to the method of Zhou et al. (2022). The total and organic selenium contents of wheat grains harvested from each plot were determined after wheat maturity. The total and organic selenium contents of spikes, leaves, stems and roots of Huamai1168 were also examined. The methods for determining total and organic selenium content are provided in **Supplementary Table S1**. The biotransformation efficiency of selenium by wheat can be calculated by the following equation:


$$\begin{array}{c} \text{Selenium Bio-conversion Rate}\\ =(\text{OrganicSelenium Content}/\text{Total Selenium Uptake})\times 100\% \end{array}$$

### 2.3 Statistical analyzes

We used Origin version 9 to draw bar graphs and SPSS version 22.0 for LSD (Least-Significant Difference) test to evaluate the differences in the target traits among the varieties. Correlation analysis (Pearson correlation) and Mantel_test (a statistical method used to assess the correlation between two distance or similarity matrices) were performed by cor and mantel_test functions in R, and results were visualized by the ggplot2, linkET, and igraph packages in R 3.6.0.

## 3 Results

### 3.1 Response of wheat grain to bio-nano-selenium

#### 3.1.1 Differences in uptake of bio-nano-selenium by wheat grain of different varieties

**Figure 1:**
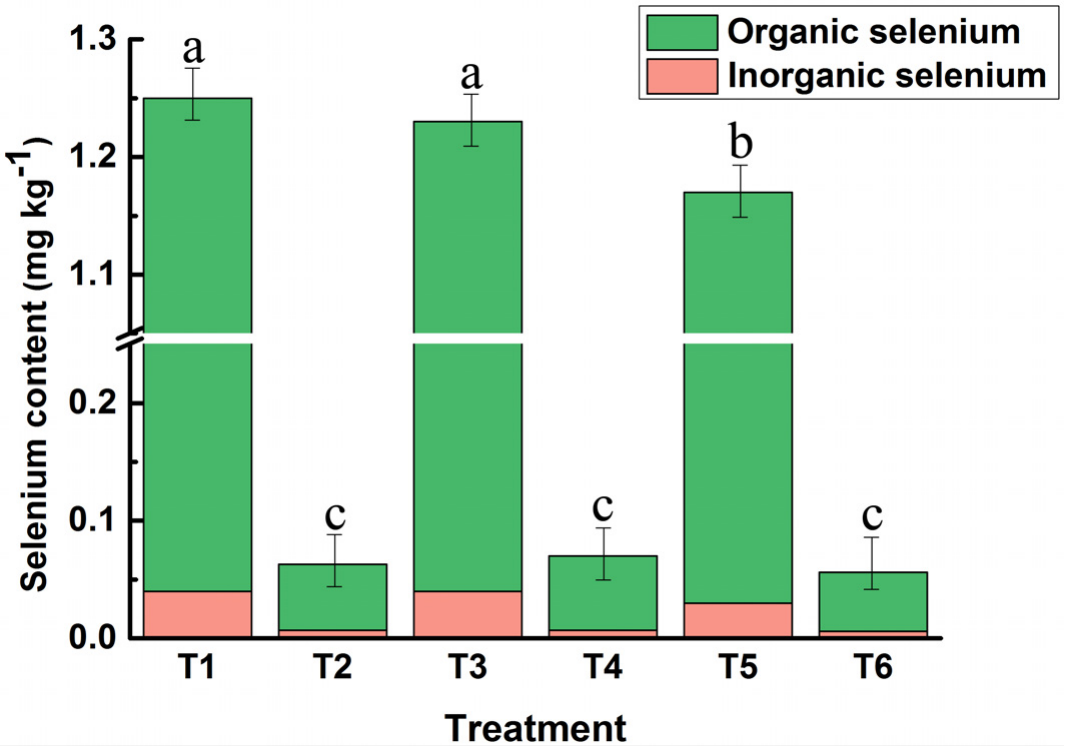
Differences in the uptake of bio-nano-selenium by the grains of the three wheat varieties. T1: Selenium-treated group of Huamai1168; T2: Control group of Huamai1168; T3: Selenium-treated group of Huamai2152; T4: Control group of Huamai2152, T5: Selenium-treated group of Wanximai0638; T6: Control group of Wanximai0638. Bars with the same letter are not significantly different between treatment groups (P < 0.05). Different colors represent different selenium levels.

Foliar spraying of bio-nano-selenium nutrient solution at the early stage of wheat flowering significantly increased seed selenium concentration in all three wheat varieties, and there were differences in the efficiency of selenium biotransformation among the varieties (**Figure 1**). Among them, the total and organic selenium content in the grains of high-gluten wheat Huamai1168 was the highest (T1), increasing by 1884.13% in total selenium and 2060.71% in organic selenium compared to the control group (T2), with the percentage of organic selenium increasing by 8.9%. The total and organic selenium content in the grains of medium-gluten wheat Huamai2152 was the second highest (T3), increasing by 1657.14% in total selenium compared to the control group (T4), with the percentage of organic selenium increasing by 1788.89%, and the percentage of organic selenium rising by 7.50%. Low gluten wheat, Wanximai0638, had the lowest total and organic selenium content in the grains (T5). Compared with the control group (T6),total selenium increased by 1989.29%, organic selenium increased by 2180.00%, and the percentage of organic selenium increased by 9.13%. The percentage of organic selenium in all three wheat varieties exceeded 96%.

#### 3.1.2 Effect of bio-nano-selenium on wheat grain morphology

**Figure 2:**
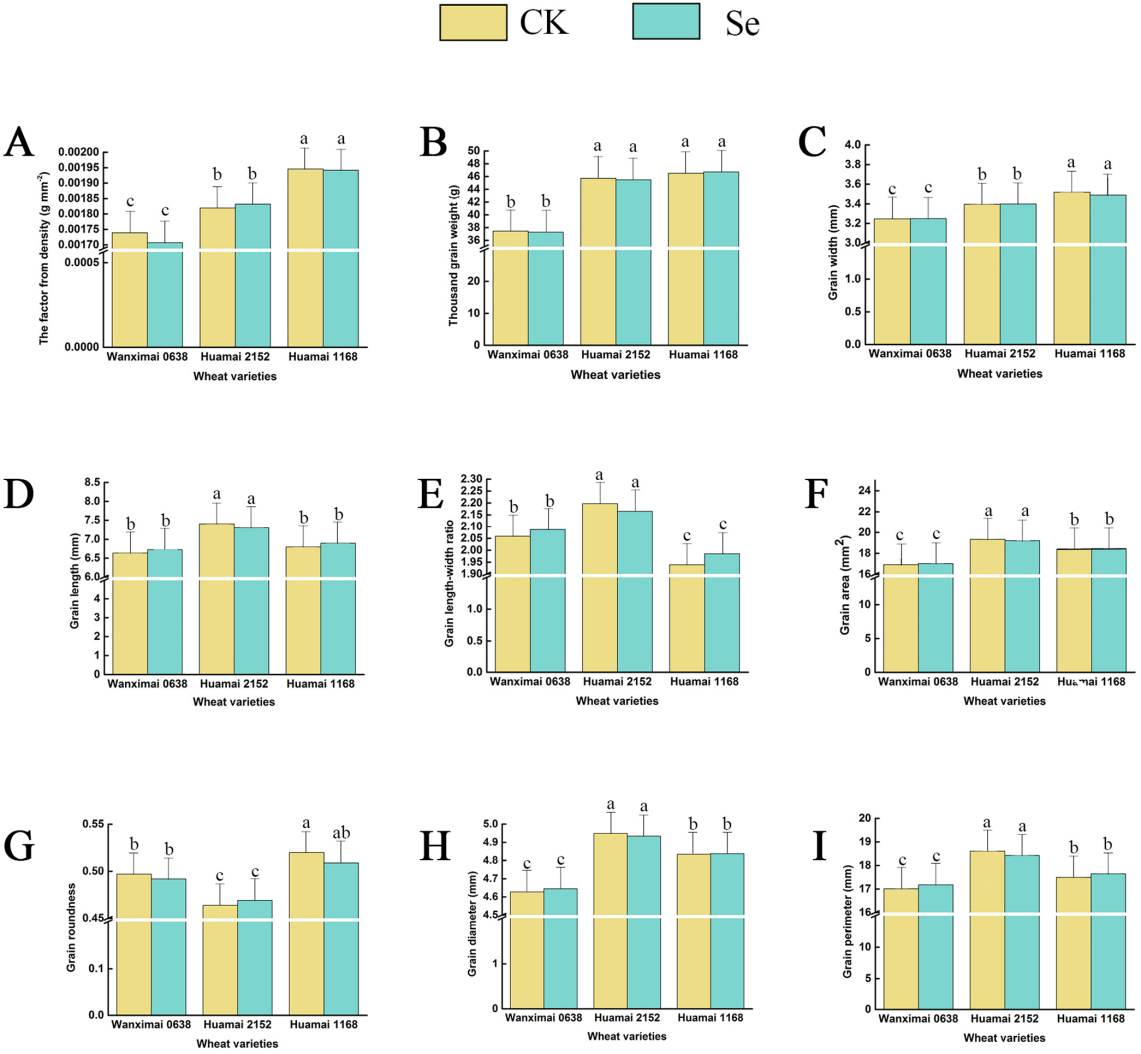
Effect of bio-nano-selenium on wheat grain morphology. **(A-I)** Comparison of two treatments across three wheat varieties for the factor from density **(A)**, thousand-grain weight **(B)**, grain width **(C)**, grain length **(D)**, grain length-width ratio **(E)**, grain area **(F)**, grain roundness **(G)**, grain diameter **(H)**, and grain perimeter **(I)**. Bars with the same letter are not significantly different between treatment groups (P < 0.05). Different colors represent different treatments.

The results of the effects of bio-nano-selenium on grain morphology-related traits in three wheat varieties are shown in **Figure 2**. After comparative analysis, we found that the exogenous supplementation of wheat with bio-nano-selenium did not significantly affect the grain morphology-related traits.

### 3.2 Response of wheat leaves to bio-nano-selenium

**Figure 3:**
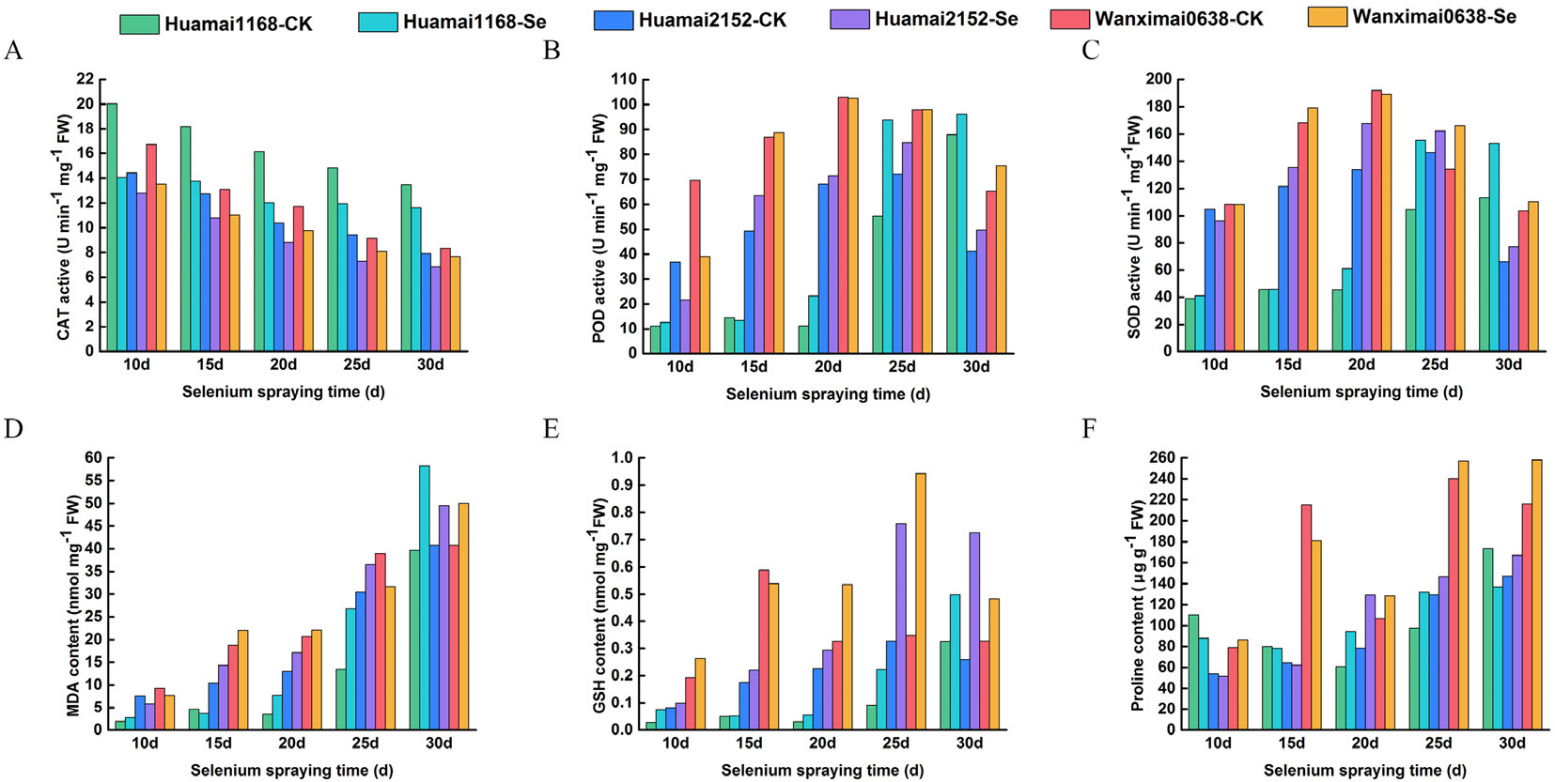
Effect of bio-nano-selenium on leaf physiological indies of different wheat varieties. **(A-F)** Comparison of selenium-treated and control groups across three varieties for CAT activity **(A)**, POD activity **(B)**, SOD activity **(C)**, MDA content **(D)**, GSH content **(E)**, and proline content **(F)**. Different colors represent different wheat varieties.

As shown in **Figure 3A**, the CAT activity of all three wheat varieties gradually declined as wheat entered the filling stage. In each time period, the CAT activity in the selenium-treated group showed varying degrees of decline compared to the control group. Huamai1168 exhibited the largest decline, with an average of 22.55% over the five periods; Huamai2152 showed the second-largest decline, with an average decrease of 15.51%; and Wanximai0638 had the smallest decline, with an average decrease of 14.12%. The results of POD activity for the three wheat varieties are shown in **Figure 3B**, which demonstrated an overall trend of increase followed by a decrease. After exogenous selenium supplementation, POD activity increased by an average of 38.42% in Huamai1168, increased by 6.09% in Huamai2152, and decreased by5.26% in Wanximai0638 compared to the control. **Figure 3C** shows the results for SOD activity, where all three wheat varieties exhibited an increasing followed by a decreasing trend over the five periods. The SOD activities in the selenium-treated groups were higher than that in the control, in the order: Huamai1168 > Huamai2152 > Wanximai0638. MDA content gradually increased as wheat filling (**Figure 3D**), and was enhanced to varying degrees after selenium treatment. The pattern of change in MDA content was more consistent with SOD activity. **Figure 3E** presents the GSH content, which first increased and then decreased over the filling period. GSH content was enhanced after foliar selenium supplementation, and the enhancement effect was proportional to the gluten content of the wheat varieties. The results for proline content are shown in **Figure 3F**. The proline content of all three wheat varieties increased over the grouting period, with an overall improvement in proline content following bio-nano-selenium spraying. Among the varieties, Huamai2152 showed the most improvement, while Huamai1168 showed the least.

### 3.3 Effect of bio-nano-selenium on agronomic traits of wheat

The results of agronomic traits examined in three wheat varieties after foliar spraying of the bio-nano-selenium nutrient solution are shown in **Table 1**. After comparison, we found that bio-nano-selenium had positive effects on Plant height (PH), number of effective spike (ES), panicle neck length of the main spike (LFPMS), rachis internode length of main spike (RLMS) and number of spikelets per plant (NSPP). The greatest effects were observed on RLMS, ES and NSPP, which increased by 21.95%, 19.03% and 17.82%, respectively, while the effects on PH and LFPMS were smaller, with increased of 8.13% and 3.82%, respectively. Meanwhile, we found that bio-nano-selenium decreased three traits: length of main spike (LMS), spikelets on main spike (SMS), and grain number per plant (GNP), by 2.16%, 1.16% and 1.13%, respectively.

**Table 1:** Effect of bio-nano-selenium on agronomic traits of wheat.

Trait	CK							Se						
	Max	Min	Mean	SD	CV (%)	Sk	Ku	Max	Min	Mean	SD	CV (%)	Sk	Ku
PH (cm)	75.80	57.90	65.70	1.92	2.92	0.32	-0.26	85.70	56.60	71.04	3.17	4.46	-0.34	-0.26
ES	4.00	2.00	2.89	0.60	20.76	-0.02	1.13	6.00	2.00	3.44	0.44	12.79	0.97	0.30
LMS (cm)	10.80	8.60	9.74	0.23	2.36	-0.23	-0.12	10.80	8.20	9.53	0.28	2.94	0.25	-0.12
LFPMS (cm)	25.40	15.70	20.17	0.92	4.56	0.44	0.91	26.80	15.60	20.94	1.29	6.16	0.07	-1.26
RLMS (cm)	6.40	2.85	4.42	0.45	10.18	0.62	-1.52	8.40	2.10	5.39	0.82	15.21	-0.17	-1.65
SMS	20.00	18.00	19.00	0.29	1.53	0.00	-1.72	22.00	15.00	18.78	0.68	3.62	-0.40	0.44
NSPP	65.00	33.00	51.11	3.63	7.10	-0.41	-0.66	99.00	35.00	60.22	7.12	11.82	0.78	-0.49
GNP	128.00	78.00	108.33	5.12	4.73	-0.73	0.67	175.00	32.00	107.11	17.45	16.29	-0.22	-1.55

### 3.4 Bio-nano-selenium uptake and transport in wheat

#### 3.4.1 Differences in selenium content among different wheat parts

**Figure 4:**
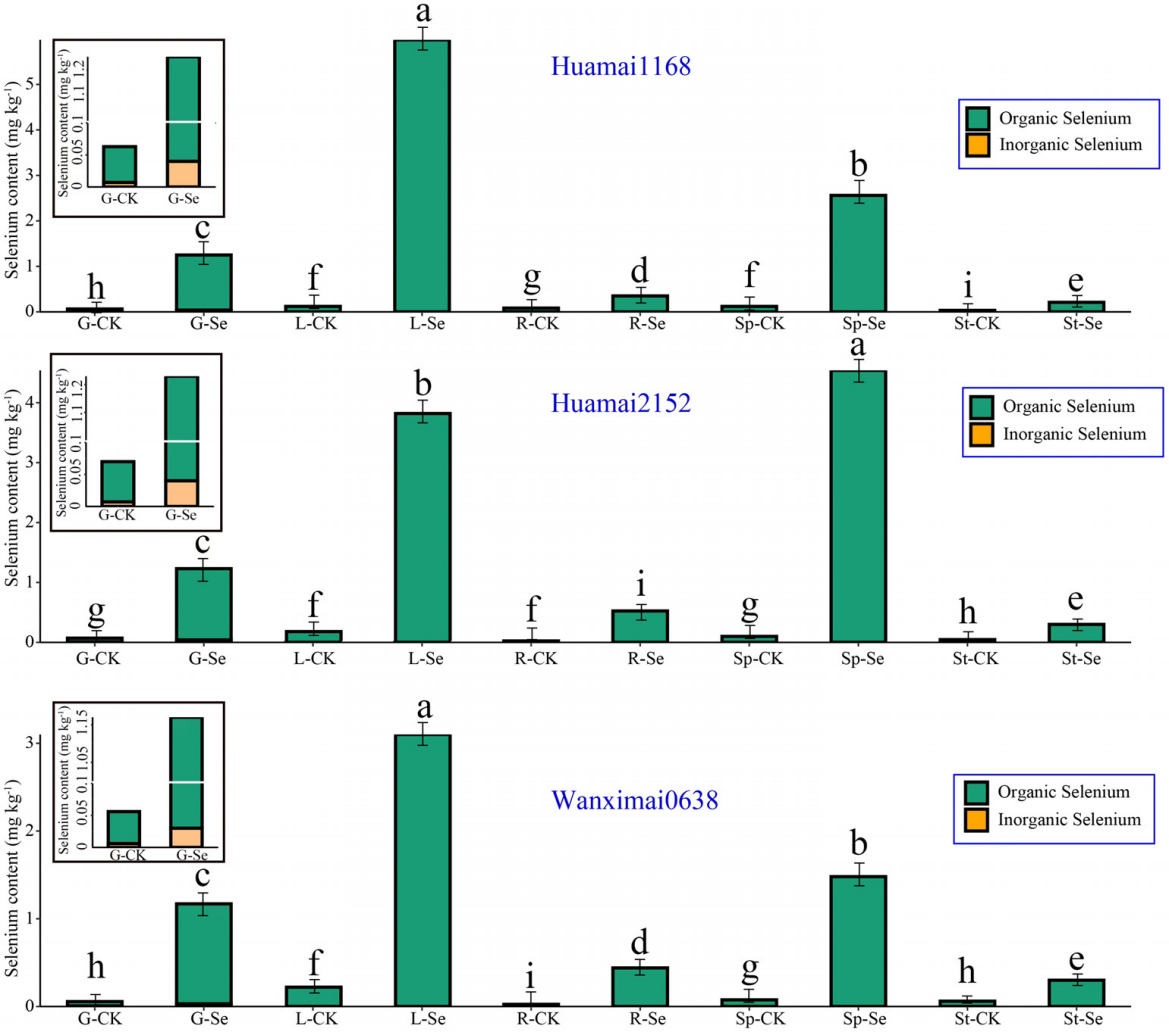
Differences in the uptake of bio-nano-selenium by various parts of wheat. In **Figure 4**, the following abbreviations represent the respective groups: G-CK: grain control group; G-Se: grain selenium treatment group; Sp-CK: spike control group; Sp-Se: spike selenium treatment group; L-CK: leaf control group; L-Se: leaf selenium-treated group; St-CK: stem control group; St-Se, stem selenium-treated group; R-CK: root control group; R-Se: root selenium-treated group. Bars with the same letter are not significantly different between treatment groups (P < 0.05).

We measured the selenium concentration in different parts of wheat, and the results are shown in **Figure 4**. In the control group, the total selenium content and organic selenium content in the wheat parts followed the order: leaf > spike > grain > stem > root. In the selenium-treated group, the total selenium content and organic selenium content in wheat parts followed the order: leaf > spike > grain > root > stem.

After foliar spraying of the bio-nano-selenium nutrient solution, the total selenium concentration in the leaves increased by an average of 2738.21%, with organic selenium content increasing by the same percentage. The total selenium concentration in the spikes increased by 2773.57%, with organic selenium content also increasing by 2773.57%. In the grains, the total selenium concentration increased by 1843.52%, and organic selenium content increased by 2009.87%. In the roots, the total selenium concentration increased by 1355.01%, with organic selenium content increasing by the same amount. The total selenium concentration in the stems increased by 493.44%, with organic selenium content also increasing by 493.44%.

#### 3.4.2 Differences in selenium content among fractions of huamai 1168

We examined the soil physicochemical indexes and selenium content of Huamai1168 before and after exogenous selenium application, and the results are shown in **Table 2**. Soil selenium content increased by 13.01% through foliar spraying of bioorganic selenium nutrient solution. It was also found that bio-organic-selenium could increase soil pH, organic matter, ammonium nitrogen, nitrate nitrogen, Ca^2+^ and Mg^2+^ content by 1.91%, 21.84%, 308.40%, 92.87%, 8.09% and 46.20%, respectively. However, its application decreased the contents hydrolytic nitrogen, effective phosphorus, fast-acting potassium and SO_4_> ^2-^ by 4.35%, 31.05%, 6.48% and 53.03%, respectively.

**Table 2:** Effect of bio-nano-selenium on basic physical and chemical properties of Huamai1168 soil.

Item	Total Selenium content (mg/kg)	pH	Organic matter (%)	Hydrolytic nitrogen(mg/kg)	Ammonium nitrogen(mg/kg)	Nitrate nitrogen (mg/kg)	Effective phosphorus(mg/kg)	Fast-acting potassium(mg/kg)	Ca^2+^ (mg/kg)	Mg^2+^ (mg/kg)	SO_4_> ^2-^(mg/kg)
Ck ^a^	0.78	6.29	4.35	161.00	7.14	39.98	118.97	147.78	18.54	7.90	469.66
Bio-nano-selenium	0.89	6.41	5.30	154.00	29.16	77.11	82.03	138.20	20.04	11.55	220.58
Increase	13.01%	1.91%	21.84%	-4.35%	308.40%	92.87%	-31.05%	-6.48%	8.09%	46.20%	-53.03%

a CK, control.

### 3.5 Correlation analysis and mantel test

**Figure 5:**
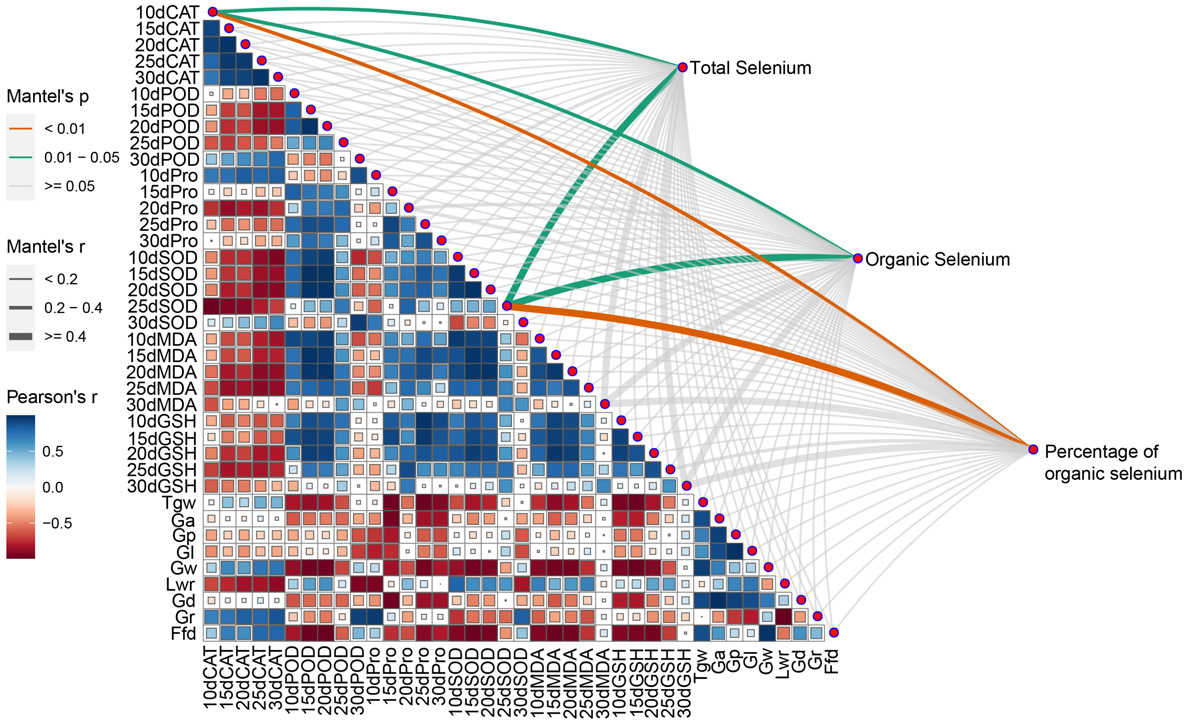
Pearson’s correlation coefficient and Mantle’s test between selenium and target traits. CAT, CAT activity; POD, POD activity; Pro, Proline content; SOD, SOD activity; MDA, MDA content; GSH, GSH content; Tgw, Thousandgrain weight; Ga, Grain area; Gp, Grain perimeter; Gl, Grain length; Gw, Grain width; Lwr, Grain length-width ratio; Gd, Grain diameter; Gr, Grain roundness; Ffd, Factor from density.

All three selenium content indicators of wheat grain were significantly and positively correlated with 10dCAT and 15dSOD. There were also negative correlations between morphological traits of the grain and antioxidant indicators in the leaves (**Figure 5**). CAT activity was negatively correlated with POD activity, Pro content, SOD activity, MDA content, GSH content, and Lwr, while it was positively correlated with Gr. POD activity was positively correlated with SOD activity, MDA content, and GSH content, but negatively correlated with Tgw, Ga, Gw, Gd and Ffd. Pro content was generally negatively correlated with grain morphology. SOD activity was positively correlated with MDA content and GSH content, but negatively correlated with Tgw, Ga, Gw, Gd and Ffd. MDA content and GSH content were negatively correlated with traits related to seed morphology. Tgw was negatively correlated with Ga, but positively correlated with Ga, Gp, Gl, Gw, Gd and Ffd. Ga was positively correlated with Gp, Gl, Gw and Gd. Gp and Gl were positively correlated with Gd and Lwr, but negatively correlated with Gr. Gw was positively correlated with Ffd and Gd. lwr was significantly negatively correlated with Gr.

## 4 Discussion

### 4.1 Effect of bio-nano-selenium on wheat plants

In our study, the selenium content of wheat was significantly and positively correlated with antioxidant enzyme activities (e.g., CAT, SOD) after exogenous selenium supplementation, suggesting that bio-nano-selenium application not only increased the selenium content of wheat but also enhanced its antioxidant defense system. However, it was also found that there may be a trade-off between selenium application and grain yield. It has been shown that selenium, as a cofactor of antioxidant enzymes, significantly enhances the antioxidant capacity of wheat and its tolerance to environmental stresses through multiple mechanisms, such as modulation of the antioxidant defense system (Alsamadany et al., 2023), enhancement of sulfur metabolism (Boldrin et al., 2016), lowering of peroxide levels (Elkelish et al., 2019), and modulation of relevant gene expression (Huang et al., 2023). These physiological mechanisms provide protection for wheat growth under adverse environmental conditions. However, during the reproductive growth stage of wheat, although selenium application enhances antioxidant enzyme activities, it may also alter the plant’s amino acid metabolism (Xiao et al., 2023), protein synthesis (Xia et al., 2020), and carbon and nitrogen metabolism (Zahedi et al., 2019), which in turn affects grain yield. Therefore, in the production of selenium-enriched wheat, rational control of the timing of selenium application is particularly important. Only by ensuring the enhancement of selenium content while optimizing both yield and quality can the best growth benefits be achieved (Wang et al., 2020).

#### 4.1.1 Differences in bio-nano-selenium uptake by wheat varieties

Selenium uptake in wheat is affected by a variety of factors, including genotype, selenium source form, selenium application techniques, and environmental factors (Xian et al., 2023). Among them, the selection of wheat varieties has an important impact on crop yield and quality (Xiao et al., 2022). In this study, we compared the selenium uptake of three representative high-, medium-, and low-gluten wheat varieties in China using bio-nano-selenium. The results showed that bio-nano selenium significantly increased the total and organic selenium content in wheat kernels by up to 19.27-fold compared with the control. The highest selenium content was found in the kernels of the high-gluten wheat variety Huamai1168, followed by the medium-gluten wheat variety Huamai2152, and the lowest selenium content was found in the kernels of the low-gluten wheat variety Wanximai 0638 (**Figure 1**). This finding is consistent with previous studies that have shown significant differences in selenium uptake among different wheat varieties (Wang et al., 2021). Wheat varieties with high gluten content may have a greater capacity for selenium uptake and accumulation because their protein synthesis process is more active and requires more sulfur amino acids, which play a crucial role in selenium uptake and transport (Trippe and Pilon-Smits, 2021). Therefore, wheat varieties with higher protein content have a greater capacity to accumulate selenium.

#### 4.1.2 Differences in bio-nano-selenium uptake by wheat varieties

Selenium is an element with a narrow safety threshold, and the safest and most effective dosage for exogenous supplementation of inorganic selenium to wheat is 15 g ha^-1^ (Di et al., 2023). The application of bio-nano-selenium in this study reached 23 g ha^-1^, which suggests that bio-nano-selenium is safer than inorganic selenium in agricultural production (Nikam et al., 2022). About 63% of wheat in China is selenium-deficient, and the average concentration of selenium in wheat grains is only 64.6 μg kg^-1^ (Li et al., 2020). In contrast, the average selenium content of the grains of the three wheat varieties in this study was 1,220 μg kg^-1^ (**Figure 1**). Based on the calculation that the total selenium content of selenium-enriched wheat kernels is reduced by 35% after processing into pasta (Cheng et al., 2022), our selenium-enriched wheat, once converted into pasta, provides a selenium content of approximately 793 μg kg^-1^. The Chinese Nutrition Society recommends an average daily selenium intake of 60-400 μg per person (National Health and Family Planning Commission of the People’ s Republic of China, 2017). We calculated that the daily recommended intake of our finished pasta products is 76-505 g, which can satisfy people’s daily selenium supplementation needs and aligns with the dietary habits of China’s residents.

#### 4.1.3 Differences in bio-nano-selenium uptake by wheat varieties

**Figure 6:**
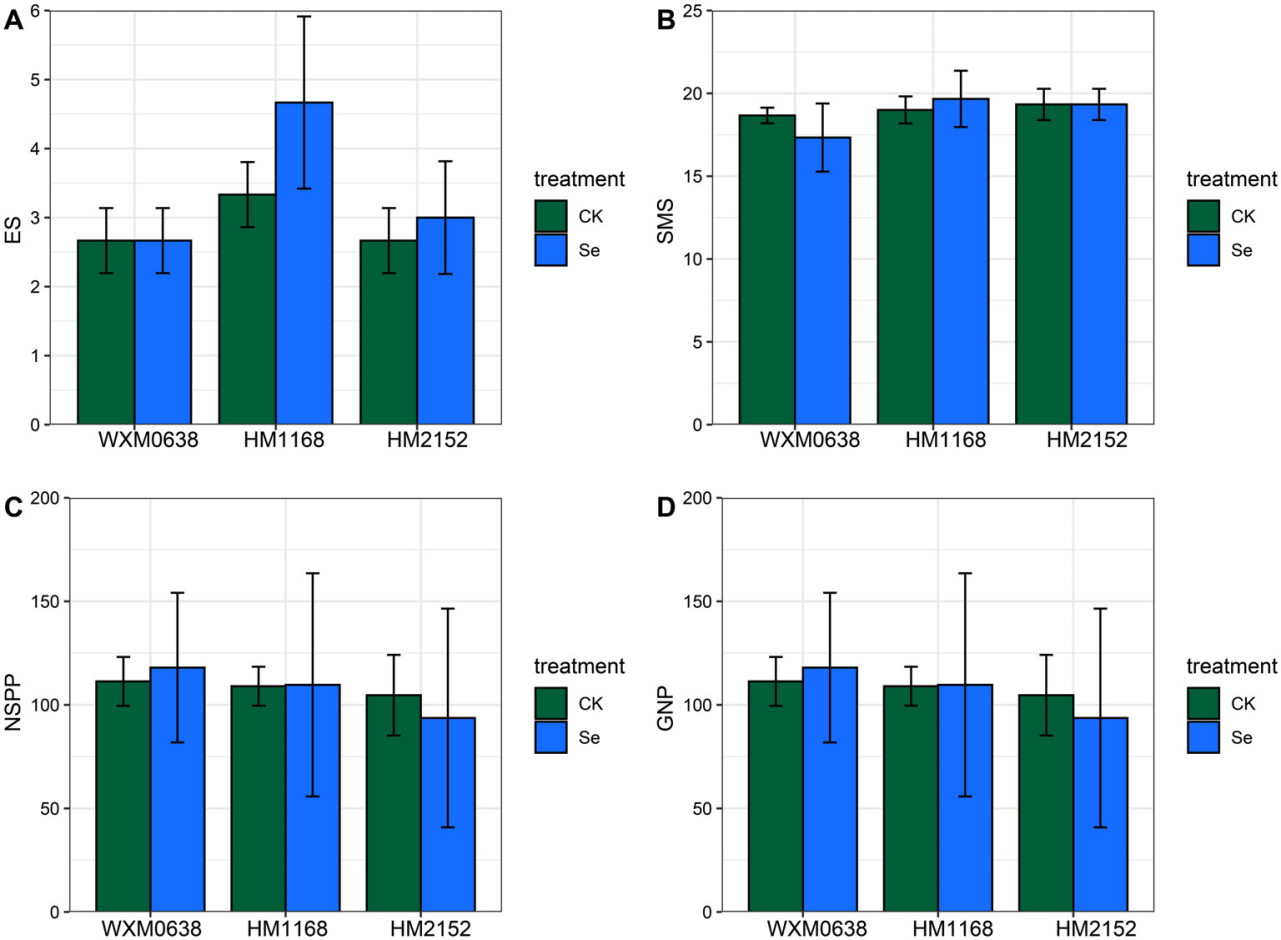
Effect of bio-nano-selenium on spike traits in three wheat varieties. **(A)** ES indicates the number of effective spikes; **(B)** SMS indicates the spikelets on the main spike; **(C)** NSPP indicates the number of spikelets per plant; **(D)** GNP indicates the number of grains per plant. WXM0638 refers to the wheat variety Wanximai 0638; HM1168 refers to the wheat variety Huamai 1168; and HM2152 refers to the wheat variety Huamai 2152. Different colors represent different treatments: CK represents the blank control group, and Se represents the selenium treatment group.

A large number of studies have shown that appropriate supplementation of selenium can increase crop yield (Delaqua et al., 2021; Huang et al., 2023a; Pourebrahimi et al., 2023; Tao et al., 2023; Zahedi et al., 2019). In our study, we compared the thousand-grain weight of wheat and its seed morphology but did not observe aa significant promoting or inhibiting effect from praying bio-nano-selenium on these indicators (**Figure 2**). Wheat yield is influenced by three main factors: the number of plants per unit area, the number of spikes per spike, and the thousand-grain weight (Clark et al., 2023). Therefore, we also tested the effective spike number, the number of spikelets in the main spike, the number of spikelets per plant, and the number of solid grains per plant, but found that selenium did not have a significant effect on any of these spike traits (**Figure 6**). We speculate that this may be related to thetime of selenium spraying, as these traits are largely determined during the early stages of the wheat’s growth period.

#### 4.1.4 Effect of bio-nano-selenium on physiological indicators of wheat leaves

In our study, selenium treatment significantly increased the activities of catalase (CAT) and superoxide dismutase (SOD) in wheat leaves, which is consistent with previous studies (Chen et al., 2023). Selenium acts as a component of glutathione peroxidase (GSH-PX) and is involved in its synthesis, which in turn enhances the antioxidant capacity of wheat (Weaver and Skoyta, 2022). Additionally, it has been shown that the application of exogenous selenium during the reproductive growth stage of wheat contributes to improved grain yield and stress tolerance, but the exact mechanism still requires further physiological studies (Huang et al., 2023; Liu et al., 2023).

For the changes in MDA (malondialdehyde) content, we found that MDA content was generally higher in the selenium-treated group than in the control group. The accumulation of MDA in wheat leaves in the selenium-treated group followed a trend consistent with the plant’s senescence process (Elkelish et al., 2019). Although selenium enhances the activity of antioxidant enzymes, it may indirectly contribute to the accumulation of MDA by altering the plant’s physiological metabolism, which, in turn, leads to an increase in cell membrane permeability (Liu et al., 2023).

Catalase (CAT) activity exhibits dynamic changes during the growth of wheat (Bhardwaj et al., 2021). In the present study, the reasons for the decrease in CAT activity after selenium spraying in wheat may be multifaceted, including the direct effect of selenium fertilizer on CAT activity, regulation of homeostasis with the antioxidant system of wheat, the growth stage and physiological status of the plant, and possible biochemical reactions (Lan et al., 2019). The specific mechanism behind this phenomenon still requires further experimental studies to gain a deeper understanding of its physiological basis.

Free proline (Pro) is an important osmoregulatory compound for plants in response to adversity, such as drought. Under adverse conditions like drought, plants reduce cellular osmotic potential, maintain cellular water, and increase drought tolerance by enhancing the synthesis of free proline (Kaur and Asthir, 2015). Selenium has been found to significantly increase free proline content in plants (Liu et al., 2023), a result consistent with our findings. We speculate that selenium may promote proline synthesis by regulating the activities of enzymes involved in free proline synthesis, such as P5CS and P5CR (Jamshidi et al., 2022). Additionally, selenium may indirectly affect free proline accumulation by regulating hormone levels and signaling pathways in plants (Chao et al., 2022). Therefore, the role of selenium in wheat is not limited to enhancing antioxidant capacity; it may also improve plant adaptability to adversity by regulating physiological metabolism and hormonal signaling.

### 4.2 Uptake and translocation of bio-nano-selenium in wheat

Bio-nanomaterials, especially nano-selenium, significantly contribute to overall plant growth and development by promoting root growth, enhancing water and nutrient uptake, and boosting antioxidant capacity (Samynathan et al., 2023). In this study, we examined the selenium content in various parts of wheat after maturation and found that wheat absorbed selenium primarily through the roots. Selenium was mainly accumulated in the spikes, while the selenium content in the seeds was relatively low, with the selenium content in the spikes being 1.905 times higher than that in the seeds. After foliar exogenous supplementation with bio-nano-selenium, selenium was primarily accumulated in the leaves, and the selenium accumulation capacity in the seeds increased compared to the control. Specifically, the selenium content in the spikes was 2.048 times higher than that in the seeds, and the efficiency increased by 7.5%. We hypothesized that foliar spraying of selenium enables direct uptake by wheat leaves, with subsequent translocation to the seed kernels through the phloem (Xia et al., 2020), thus shortening the process of selenium uptake from the roots. In addition, bioactive molecules attached to the surface of nanoparticles, such as polysaccharides, peptides, and phytohormones, promoted root development by enhancing interactions with plant cells (Yuan et al., 2022). These bioactive molecules also helped the plant efficiently maintain the flow of water and nutrients by regulating ionic balance and osmotic adjustments within the cells, which in turn improved photosynthetic efficiency and the plant’s resilience (Rasheed et al., 2024). Nodes in wheat play an important role in the transport and distribution of mineral elements (Xiao et al., 2024), and foliar spraying of nano-selenium may further accelerate the transport of selenium from leaves to seeds by promoting the expression or activity of transporter proteins in the nodes. However, the specific mechanism of action still needs to be verified by further experiments.

### 4.3 Effect of application of bio-nano-selenium on soil physic-chemical indicators

In this study, we also preliminarily investigated the effects of foliar spraying of bio-nano-selenium on soil physicochemical indicators in wheat. Some studies have shown that the application of nano-selenium can improve soil quality (Samynathan et al., 2023), which is consistent with our findings. Bio-nano-selenium resulted in a significant increase in ammonium nitrogen (308.40%) and nitrate nitrogen (92.87%), which may be attributed to the fact that bio-nano-selenium enhances the nitrification capacity of the soil by promoting the growth and reproduction of microorganisms such as nitrifying bacteria, thus accelerating the conversion of ammonium nitrogen to nitrate nitrogen (Liu J. et al., 2021). Meanwhile, the pH of the selenium-treated group increased by 1.91%, which may be due to changes in microbial activities or the interaction of selenium with other soil constituents, altered the soil’s chemical composition (Zhou et al., 2020). Soil organic matter increased by 21.84%, which we speculate may be due to bio-nano-selenium promoting plant growth, positively influencing microbial activity and decomposition processes, and thus increasing the amount of organic matter in the soil (Liu et al., 2021). In addition, calcium and magnesium in the soil increased by 8.09% and 46.20%, respectively. We speculate that bio-nano-selenium increased the biomass and activity of wheat roots, which caused the root system to release more organic acids, dissolving more calcium and magnesium from the soil particles and increasing their availability in the soil (Samynathan et al., 2023). However, the exact mechanism needs further investigation. On the other hand, selenium application also led to decreases in some soil physicochemical indices. In our study, hydrolyzed nitrogen in the soil of selenium-treated group decreased by 4.35%, effective phosphorus decreased by 31.05%, and quick-acting potassium decreased by 6.48%. We hypothesize that this is because bio-nano-selenium promotes plant growth, increasing the plant’s demand for nitrogen, phosphorus, and potassium, which in turn reduces their content in the soil (Samynathan et al., 2023). SO_4_> ^2-^ was reduced by 53.03%, which may be due to vigorous plant growth after selenium application, leading to increased uptake of SO_4_> ^2-^, thus reducing sulphate content in the soil (Samynathan et al., 2023). Alternatively,selenium application may stimulate the activity of soil microorganisms, particularly those involved in sulfur cycling, accelerating the conversion and metabolism of sulfur and consuming sulfate in the soil (Zhuang et al., 2024). The specific mechanism still requires further experimental verification.

Although foliar spraying of bio-nano selenium shortened the process of selenium uptake by wheat roots and enhanced seed uptake efficiency in this study, the translocation and uptake of bio-nano-selenium in wheat plants also affected soil physiological indices through the root system. In addition to promoting selenium uptake and transformation in wheat, bio-nano-selenium also improved the uptake and utilization of other nutrients in the soil. This not only enhanced the biotransformation efficiency of selenium in wheat and its effective accumulation in the plant, but also had a positive overall effect on the healthy growth of wheat.

## 5 Conclusions

The present study showed that bio-nano selenium significantly increased the selenium content in wheat grains, and the selenium uptake capacity varied among different wheat varieties. High-gluten wheat (Huamai1168) showed the most significant selenium uptake and organic selenium accumulation, while low-gluten wheat (Wanximai0638) exhibited the lowest selenium accumulation. In addition, selenium treatment enhanced the antioxidant enzyme activity of wheat, improved plant stress tolerance, and improved the physicochemical properties of the soil by promoting the increase of nutrients such as ammonium nitrogen and nitrate nitrogen, while decreasing hydrolyzed nitrogen, effective phosphorus, and potassium. In summary, bio-nano selenium can not only effectively increase the selenium content of wheat and enhance its resistance, but also improve soil quality as an ideal selenium source for the production of selenium-enriched wheat, especially in high-gluten wheat varieties, which showed more significant advantages.

## Data Availability

The original contributions presented in the study are included in the article/**Supplementary Material**. Further inquiries can be directed to the corresponding authors.
