# Supplementary material for: Effects of bio-nano-selenium on wheat grain morphology, selenium transport enrichment and antioxidant enzyme activities
Source: Front Plant Sci. 2025 Mar 5;16:1516005. doi: 10.3389/fpls.2025.1516005 (PMC11920120; doi:10.3389/fpls.2025.1516005)
Supplement: Supplementary file 1 [file Table1.docx]

Supplementary Table S1 Determination methods for target traits

| Traits | | Units | Abbr. | Method |
| --- | --- | --- | --- | --- |
| Leaf Physiological Indicators | Catalase activity | U min^-1^ mg^-1^ FW | CAT | Sample Preparation: Leaves were collected from the wheat plants at specific intervals after foliar spraying of the bio-nano-selenium solution. These samples were immediately placed in ice packs and stored at 4°C until analysis.  CAT Activity Assay: Catalase activity was measured by a spectrophotometric method, which involves monitoring the decomposition of hydrogen peroxide (H₂O₂) at 240 nm. The decrease in absorbance at 240 nm is recorded over time, and the activity is expressed in terms of the amount of enzyme required to decompose a given amount of H₂O₂ per unit time. |
|  | Peroxidase activity | U min^-1^ mg^-1^ FW | POD | Sample Collection: Wheat leaf samples were collected at various time points (10th, 15th, 20th, 25th, and 30th days) after the application of the bio-nano-selenium nutrient solution.  POD Activity Assay: The activity of POD was determined using a spectrophotometric method. This involves measuring the rate of decomposition of hydrogen peroxide (H₂O₂) in the presence of guaiacol, which is converted into a brown-colored compound. Enzyme activity was assessed by monitoring the increase in absorbance at 470 nm and the results were expressed in units of POD activity per gram of fresh weight of leaf tissue. |
|  | Superoxide dismutase activity | U min^-1^ mg^-1^ FW | SOD | Sample Collection: Wheat leaf samples are collected at multiple time points after selenium treatment. These are placed in ice immediately after collection to preserve enzyme activity.  Determination of SOD activity: SOD activity is usually measured spectrophotometrically and the inhibition of NBT reduction is monitored by measuring absorbance at 560 nm. The inhibition of NBT reduction is monitored by measuring the absorbance at 560 nm; the higher the SOD activity, the stronger the inhibition of NBT reduction. |
|  | Malondialdehyde content | nmol mg^-1^ FW | MDA | Sample Collection: Wheat leaves are collected at specific intervals after the bio-nano-selenium treatment, placed on ice immediately, and stored at 4°C until analysis.  MDA Detection Assay: The MDA content is determined using the thiobarbituric acid (TBA) reaction. In this method, the leaf tissue is homogenized in a solution that reacts with MDA to form a colored compound. The intensity of this color is measured spectrophotometrically at 532 nm, and the absorbance value is used to quantify MDA. |
|  | Reduced glutathione content | nmol mg^-1^ FW | GSH | Sample Preparation: Wheat leaves are collected from the experimental plots at specific intervals after bio-nano-selenium treatment, placed in ice packs, and stored at 4°C until analysis.  GSH Detection Assay: The GSH content is determined using a spectrophotometric method. This assay involves reacting GSH with a chromogenic reagent, such as 5,5'-dithiobis-(2-nitrobenzoic acid) (DTNB), also known as Ellman's reagent. The reaction produces a yellow-colored product (TNB), which can be quantified by measuring absorbance at 412 nm. The GSH content is then calculated based on a standard curve of known GSH concentrations. |
|  | Proline content | μg g^-1^ FW | Pro | Sample Collection: Wheat leaves are collected at various time intervals after the selenium treatment. These samples are immediately placed on ice and stored at 4°C until analysis.  Proline Detection Assay: The proline content is measured using the acid ninhydrin method. The leaves are homogenized in sulfosalicylic acid, and the homogenate is filtered. The filtrate is reacted with acid ninhydrin and glacial acetic acid at a high temperature (usually 100°C) for about an hour. After cooling, toluene is added, and the chromophore containing proline is separated. The intensity of the color is measured spectrophotometrically at 520 nm, and the proline content is quantified against a standard curve of known proline concentrations. |
| Grain morphological traits | Plant heigh | cm | PH | Sample Collection: After the bio-nano-selenium treatment, fully matured wheat plants are selected for measurement. Typically, 10 plants are randomly chosen from each experimental plot.  Measurement Process: The height of each plant is measured from the soil surface to the top of the main spike (excluding any awns |
|  | Effective panicle |  | ES | Sample Selection: Once the wheat plants are fully matured, a total of 10 plants are randomly selected from each experimental plot.  Effective Panicle Count: The effective panicles (or spikes) are counted by identifying the number of spikes per plant that bear mature, filled grains. Panicles that do not bear grains or are underdeveloped are not considered effective. |
|  | Length of main spike | cm | LMS | Sample Selection: At full maturity, wheat plants are harvested, and 10 plants are randomly selected from each experimental plot.  Measurement Process: The main spike of each selected plant is identified. Using a ruler or measuring tape, the length of the main spike is measured from the base (where the spike attaches to the stem) to the tip of the spike (excluding the awns, if present). |
|  | Panicle neck length of main spike | cm | LFPMS | Sample Selection: After the wheat has fully matured, a group of plants is randomly selected from the experimental plot, usually 10 plants from each plot.  Measurement Process: The panicle neck length refers to the length of the stem between the flag leaf node and the base of the main spike. Using a ruler or caliper, this distance is measured in centimeters (cm). |
|  | Rachis intemode length of main spike | cm | RLMS | Sample Selection: Wheat plants are selected at full maturity, typically 10 plants from each plot.  Measurement Process: The rachis internode length refers to the length between two successive nodes along the rachis (the central axis of the spike). Using a ruler or caliper, the internode length is measured from the base to the tip of the main spike. |
|  | Spikelets on main spike |  | SMS | Sample Selection: At full maturity, wheat plants are harvested, and 10 plants are randomly chosen from each experimental plot for measurement.  Counting Spikelets: The spikelets on the main spike of each plant are manually counted. Spikelets are the small flower clusters arranged along the rachis of the main spike. The number of spikelets that are fully developed and capable of bearing grains are counted for each spike. |
|  | Number of spikelets per plant |  | NSPP | Sample Selection: At full maturity, select 10 wheat plants from each experimental plot for measurement.  Counting Spikelets: For each plant, the number of spikelets on all the spikes (not just the main spike) is counted manually. A spikelet consists of a group of flowers arranged on the spike, and the spikelets capable of bearing grains are included in the count. |
|  | Grain number per plant. |  | GNP | Sample Selection: Fully matured wheat plants are harvested, typically 10 plants from each experimental plot.  Grain Counting: For each plant, all the grains from the spikes (main spike and any additional spikes) are manually threshed. The grains are separated from the chaff, and the total number of grains produced by each plant is counted. |
| Selenium content | Total selenium content | mg kg^-1^ | / | Hydride generation atomic absorption spectrometry (HG-AAS): The samples were dried at 65 °C for 72 h, ground into fine powder and digested with HNO_3_-HClO_4_ (4:1, v/v) in a digestion oven at 180 °C for 0.5 g of sample until the solution was clear and transparent and selenium was converted to Se (IV). After cooling, the digested product was determined by hydride generation atomic absorption spectrometry (HG-AAS) for total selenium content. |
|  | Organic selenium content | mg kg^-1^ | / | Hydride generation atomic absorption spectrometry (HG-AAS): The inorganic selenium content was first determined. The samples were dryed at 65 °C for 72 h, ground into fine powder, added 0.5 g of sample powder to 20 mL of distilled water, sonicated for 30 min and water-bathed in 100 °C for 1 h to ensure adequate extraction of inorganic selenium. The samples were then centrifuged at 8500 rpm for 10 minutes at 4 °C, the supernatant was filtered through a 0.45 µm filter membrane. The selenium content was determined by HG-AAS method. The organic selenium content is the difference between the total and inorganic selenium content. |
|  | Proportion of organic selenium | % | / | Percentage of organic selenium (%) = organic selenium content (mg kg ^-1^) / total selenium content (mg kg ^-1^) × 100% |
